# Prognostic nomograms for predicting long‐term overall survival in spindle cell melanoma: a population‐based study

**DOI:** 10.3389/fendo.2024.1260966

**Published:** 2024-03-20

**Authors:** Wai Chi Lau, Liying Huang, Xinkai Zheng, Wai-kit Ming, Nga Cheng Leong, Yu Tak Wong, Zhinan Yin, Hai Yu, Jun Lyu, Liehua Deng

**Affiliations:** ^1^ Department of Dermatology, The First Affiliated Hospital of Jinan University & Jinan University Institute of Dermatology, Guangzhou, China; ^2^ Department of Infectious Diseases and Public Health, Jockey Club College of Veterinary Medicine and Life Sciences, City University of Hong Kong, Hong Kong, Hong Kong SAR, China; ^3^ Department of Clinical Research, The First Affiliated Hospital of Jinan University, Guangzhou, China; ^4^ Department of Dermatology, Kiang Wu Hospital, Macau, China; ^5^ SHENZHEN BeauCare Clinic, Shenzhen, China; ^6^ Guangdong Provincial Key Laboratory of Tumor Interventional Diagnosis and Treatment, Zhuhai Institute of Translational Medicine Zhuhai People’s Hospital Affiliated with Jinan University, Zhuhai, China; ^7^ The Biomedical Translational Research Institute, Health Science Center (School of Medicine), Jinan University, Guangzhou, China; ^8^ Guangdong Provincial Key Laboratory of Traditional Chinese Medicine Informatization, Guangzhou, China; ^9^ Department of Dermatology, The Fifth Affiliated Hospital of Jinan University, Heyuan, China

**Keywords:** surveillance, epidemiology, and end results (SEER) database, nomogram, spindle cell melanoma, overall survival, SEER

## Abstract

**Background:**

There are few research findings on the survival prognosis of spindle cell melanoma (SCM), which is an unusual kind of melanoma. The purpose of this study was to develop a thorough nomogram for predicting the overall survival (OS) of patients with SCM and to assess its validity by comparing it with the conventional American Joint Committee on Cancer (AJCC) staging system.

**Methods:**

The Surveillance, Epidemiology, and End Results database was searched, and 2,015 patients with SCM were selected for the analysis. The patients were randomly divided into training (n = 1,410) and validation (n = 605) cohorts by using R software. Multivariate Cox regression was performed to identify predictive factors. A nomogram was established based on these characteristics to predict OS in SCM. The calibration curve, concordance index (C-index), area under the receiver operating characteristic curve, and decision-curve analysis were utilized to assess the accuracy and reliability of the model. The net reclassification improvement and integrated discrimination improvement were also applied in this model to evaluate its differences with the AJCC model.

**Results:**

The developed nomogram suggests that race, AJCC stage, chemotherapy status, regional node examination status, marital status, and sex have the greatest effects on OS in SCM. The nomogram had a higher C-index than the AJCC staging system (0.751 versus 0.633 in the training cohort and 0.747 versus 0.650 in the validation cohort). Calibration plots illustrated that the model was capable of being calibrated. These criteria demonstrated that the nomogram outperforms the AJCC staging system alone.

**Conclusion:**

The nomogram developed in this study is sufficiently reliable for forecasting the risk and prognosis of SCM, which may facilitate personalized treatment recommendations in upcoming clinical trials.

## 1 Introduction

Representing only 3–14% of all melanomas, spindle cell melanoma (SCM) is an infrequent subtype of malignant melanoma that consists of spindled neoplastic cells organized in sheets and fascicles (1–3). Since SCM can develop anywhere on the body (4), it might resemble other spindle cell tumors due to the absence of typical melanoma characteristics and the varying levels of cytological atypia that it exhibits (5). SCM is even frequently misdiagnosed on clinicopathological inspection (6, 7). Given these characteristics, there have been few investigations of this condition over the past 10 years. Most of the few trials that investigated SCM also included melanoma (8–10). Previous studies have had very small study populations due to the exceedingly low SCM incidence, and they did not perform thorough prognostic assessments (11). The current study concentrated solely on SCM analysis due to the recent advancements in SCM diagnosis technology (12–14).

Treatment plans for patients with SCM have frequently been determined using the American Joint Committee on Cancer (AJCC) staging system (15). Since the prognosis of patients with SCM is affected by various other parameters such as race, relationship status, and sex, this technique is frequently employed alone to forecast the prognoses of an entire group of patients. However, it has several significant limitations. Ignoring these important prognostic markers could reduce the accuracy of survival forecasts since the survival rates of patients at the same AJCC stage can vary markedly. To improve the accuracy of survival forecasts in patients with SCM, new prognostic methods are required due to the clinical singularity of SCM (16).

Nomograms are frequently used to assist clinicians in creating treatment plans and determining the prognosis of different cancer types, but a prognostic nomogram for SCM has never been constructed using the Surveillance, Epidemiology, and End Results (SEER) database (17). Consequently, the goal of this study was to construct a thorough nomogram that also considers demographic factors. To assess the reliability of the new prediction model, it was compared with the conventional AJCC staging system. Establishing a thorough prognostic evaluation system for SCM and validating its predictive performance were the goals of the current study (18, 19).

## 2 Methods

### 2.1 Patient selection

**Figure 1:**
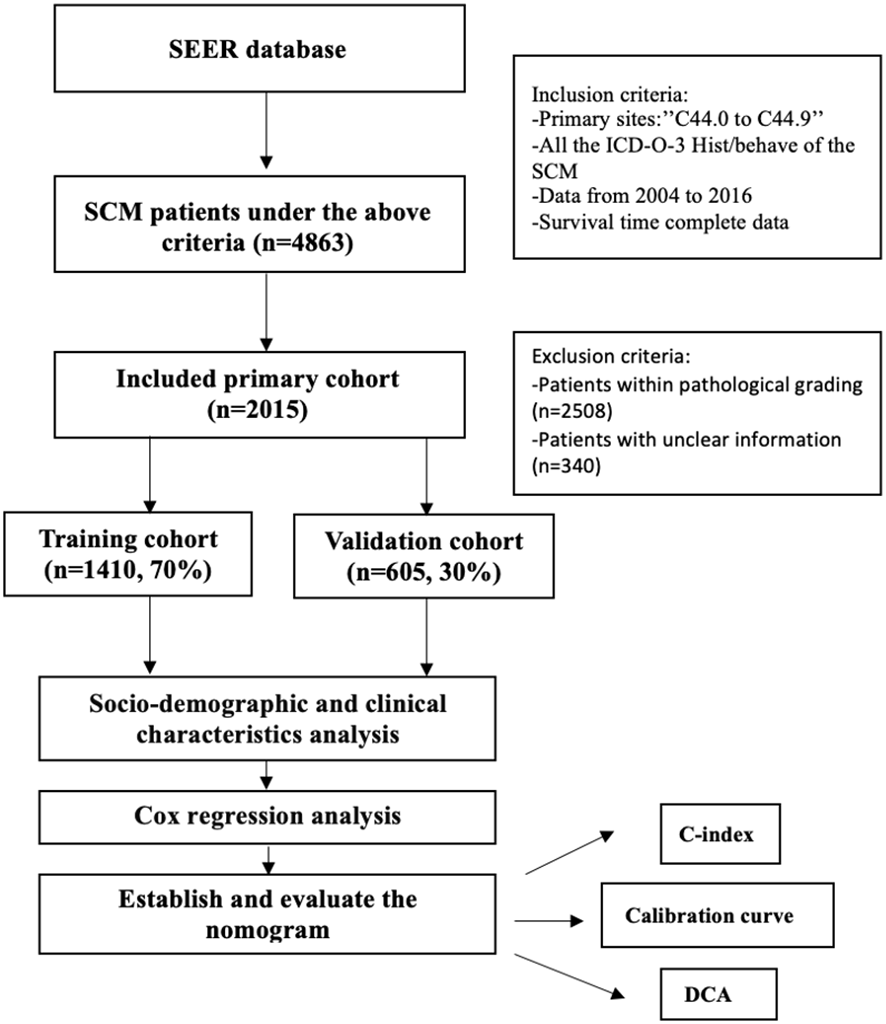
Research flowchart of selection.

We evaluated patient information from the most recent edition of the SEER database encompassing 18 registries using SEER*Stat software (version 8.3.5). We searched for the histological code 8772/3 for SCM in the third revision of the International Classification of Diseases for Oncology (ICD-O-3). The investigated factors included marital status, AJCC stage, age, race, sex, surgery status, chemotherapy status, and overall survival (OS) rate. We used the sixth version of the AJCC staging system and restricted our search to 2004–2015 because the system was established in 2004. The selection criteria were (i) age ≥18 years, (ii) ICD-O-3 code 8772, and (iii) presence of spindle cell cancer. The exclusion criteria were (i) incomplete survival data or (ii) unclear information on pathological grading. **Figure 1** depicts the screening procedure of patients in the SEER database. We randomly assigned 70% of the patients to the training cohort (n = 1,410) and 30% to the validation cohort (n = 605) for the construction and validation of the nomograms. There were 4,863 suitable cases identified in the SEER database, of which 2,015 were selected following a stringent selection process. The primary outcomes were OS, which was the primary intention from an SCM diagnosis, and the final follow-up or death regardless of cause.

### 2.2 Statistical analysis

The analyses were conducted using the seven clinical factors of age at diagnosis, marital status, race, sex, AJCC stage, chemotherapy status, and regional node examination status. Proportions were used to describe categorical data. The Cox regression model was also applied to the training cohort for variables with p < 0.05 in the multivariate Cox proportional-hazards regression analysis, indicating independent prognostic factors of SCM. A nomogram for the incidence rates of OS in patients with SCM over 3, 5, and 8 years was produced based on the forecasting model of prognostic variables.

### 2.3 Validation of the nomograms

After constructing the nomogram, we evaluated it using various indicators. The accuracy of nomograms was evaluated using the concordance index (C-index) and receiver operating characteristic (ROC) curves, and the discrimination of nomograms was verified using calibration plots. The net reclassification improvement (NRI), integrated discrimination improvement (IDI), and decision-curve analysis (DCA) were used to compare the clinical benefits and utility of the nomogram with those of the AJCC staging alone, which increased the accuracy and comprehensiveness of the comparison. NRI and IDI are two alternatives to the area under the ROC curve (AUC) for assessing risk prediction improvement and determining the usefulness of a new model. We also used DCA, which is a commonly used method for evaluating the clinical benefit of alternative models.

IBM SPSS Statistics software and R programming language and environment were used for all statistical analyses, with a bilateral probability value of p < 0.05 considered to indicate significance. R software was used to randomly split the 2,015 patients into two study cohorts at a 7:3 ratio, and the log-rank test was used to ensure that there were no significant differences between the cohorts.

## 3 Results

### 3.1 Patient characteristics

The 2,015 patients identified with SCM were randomly divided into 1,410 in the training cohort and 605 in the validation cohort. The median age was 70 years in both cohorts at the time of diagnosis. Most of the patients in both cohorts were married (60.6% and 66% in the training and validation cohorts, respectively), male (67% and 67.8%), white (98.5% and 98.9%), and at AJCC stage II (46.7% and 46.1%). Moreover, 909 (64.5%) and 390 (64.5%) patients in the training and validation cohorts received regional node examination, respectively, while 1,352 (95.9%) and 582 (96%) patients refused chemotherapy. The training and validation cohorts had median follow-up times of 74.5 and 74.6 months, respectively. The demographic and clinical characteristics did not differ significantly between the training and validation cohorts (p > 0.05). **Table 1** lists the demographics and tumor characteristics of the patients.

**Table 1:** Socio-demographic and clinical characteristics of patients in the study.

Baseline Characteristics of Patients					
	training cohort (n)	training cohort (%)	Validation cohort (n)	Validation cohort (%)	P-value
Variable	1410		605		
**Age**					0.315
20-39	66	4.6	27	4.5	
40-60	307	21.8	155	25.6	
61-80	702	49.8	286	47.3	
>80	335	23.8	137	22.6	
**Sex**					0.743
male	945	67	410	67.8	
female	465	33	195	32.2	
**Race**					0.823
white	1389	98.5	598	98.9	
black	7	0.5	2	0.3	
other	14	1	5	0.8	
**Marital status**					0.058
married	854	60.6	399	66	
unmarried	428	30.3	129	21.3	
other	128	9.1	77	12.7	
**AJCC stage**					0.609
I	487	34.5	200	33.1	
II	659	46.7	279	46.1	
III	159	11.3	71	11.7	
IV	105	7.5	55	9.1	
**Regional node examined**					0.998
no/unknow	501	35.5	215	35.5	
yes	909	64.5	390	64.5	
**Chemotderapy**					0.879
no/unknow	1352	95.9	581	96	
yes	58	4.1	24	4	

### 3.2 Variable screening

The model comprised age at diagnosis, sex, race, marital status, AJCC stage, regional node examination status, and chemotherapy status, based on the stepwise regression results. The following variables were significant in the multivariate analysis: age at diagnosis of 41–60 years (HR = 2.078, p < 0.005 versus 20–40 years), age at diagnosis of 61–80 years (HR = 5.608, p < 0.001 versus 20–40 years), age at diagnosis of >80 years (HR = 13.342, p < 0.001 versus 20–40 years), female sex (HR = 0.793, p < 0.05 versus male), black (HR = 4.144, p < 0.001 versus white), other race (HR = 1.145, p > 0.05 versus white), unmarried (HR = 1.291, p < 0.05 versus married), other marital status such as separated/divorced/widowed (HR = 1.395, p < 0.01), AJCC stage II (HR = 1.682, p < 0.001 versus stage I), AJCC stage III (HR = 3.188, p < 0.001 versus stage I), AJCC stage IV (HR = 4.701, p < 0.001 versus stage I), regional node examination (HR = 0.617, p < 0.001 versus no/unknown regional node examination), and received chemotherapy (HR = 2.072, p < 0.001 versus no/unknown chemotherapy status). **Table 2** lists the results of the multivariate Cox regression analysis.

**Table 2:** Selected variables by multivariable Cox regression analysis.

Multivariate Cox Analysis			
Prognostic factors	HR^a^	95%CI^b^	P-value
Age			
20-39	reference		
40-60	2.078	1.112-3.884	0.022
61-80	5.608	8.056-10.292	<.0001
>80	13.342	7.215-24.675	<.0001
Sex			
male	reference	–	
female	0.782	0.685-0.917	0.002
Race			
white	reference	–	
black	4.235	1.848-9.296	0.001
other	1.479	0.792-2.636	0.231
Marital status			
married	reference	–	
unmarried	1.291	1.042-1.601	0.02
other	1.395	1.201-1.620	<.0001
AJCC stage			
I	reference	–	
II	1.682	1.436-1.970	<.0001
III	3.188	2.548-3.989	<.0001
IV	4.701	3.680-6.005	<.0001
Regional node examined			
no/unknow	reference	–	
yes	0.617	0.537-0.708	<.0001
Chemotherapy			
no/unknow	reference	–	
yes	2.072	1.548-2.773	<.0001

a HR, hazard ratio; ^b^CI, confidence interval.

### 3.3 Prognostic nomograms for OS

A nomogram was constructed based on the HRs of selected variables that covered all the main independent factors for predicting the 3-, 5-, and 8-year OS rates in the training cohort. The total score is obtained by summing the individual scores of the nomogram, and a vertical line is drawn down from the total-points row to indicate the likelihoods of survival at 3, 5, and 8 years. The factor that provided the strongest prediction of prognosis was age, followed by AJCC stage, race, chemotherapy status, sex, and regional node examination status.

### 3.4 Performance of the nomograms

The C-indexes for the OS nomogram were 0.751 and 0.747 in the training and validation cohorts, respectively. The AUC values for the training cohort (0.784, 0.788, and 0.816 for 3-, 5-, and 8-year OS, respectively) and validation cohort (0.785, 0.8, and 0.807) suggested that the model had strong discriminative ability. The OS nomogram calibration plots demonstrated that the training and validation cohorts predicted 3-, 5-, and 8-year survival probabilities that were remarkably like the actual data.

The NRI values for the 3-, 5-, and 8-year OS probabilities were 0.625 (95% CI = 0.495–0.743), 0.680 (95% CI = 0.576–0.803), and 0.815 (95% CI = 0.710–0.946), respectively, in the training cohort, and 0.618 (95% CI = 0.457–0.868), 0.742 (95% CI = 0.536–0.914), and 0.771 (95% CI = 0.563-0.941) in the validation cohort. The corresponding IDI values for the 3-, 5-, and 8-year OS probabilities were 0.151, 0.183, and 0.207, respectively (p < 0.001), in the training cohort, and 0.131, 0.166, and 0.190 (p < 0.001) in the validation cohort. The NRI and IDI indices were both nonzero and negative, indicating that our model outperforms the AJCC staging system in terms of discrimination ability.

The 3-, 5-, and 8-year DCA curves of both the training and validation cohorts were improved over those for the AJCC staging system.

## 4 Discussion

SCM is an uncommon histological variant of melanoma, with reports of its prevalence among all melanoma cases ranging from 3% to 14% (2). SCM is difficult to diagnose, and so understanding its clinical features and histopathological indicators is crucial to making the correct clinical decisions (3, 8). However, SCM is extraordinarily rare, and thus its clinical and prognostic aspects are not yet thoroughly understood (13). Since SCM has distinct pathological features and prognosis prediction models, previous investigations of SCM have been insufficient and have frequently classified SCM as general melanoma (3). The prevalence of SCM has recently increased and its diagnostic technology has improved, demonstrating the increasing significance of evaluating its clinical prognosis. SCM prognoses are currently not sufficiently accurate, and there is a lack of comprehensive and simple support research for this disease (12).

An SCM-specific nomogram needs to be constructed to provide clinical personnel with more-precise prediction models due to the unique clinical aspects of SCM. Using clinical information from the SEER database, we constructed an effective predictive nomogram for patients with SCM in this study. Nomograms are more commonly utilized than the conventional AJCC staging system in oncology and medicine for prognosis predictions and to meet the needs of clinical personnel to provide patients with tailored therapies. Age at diagnosis, sex, race, marital status, AJCC stage, regional node examination status, and chemotherapy status were all related to SCM prognosis according to the multivariate Cox regression analysis performed in the current study.

Age, sex, and race seem to be significant prognostic factors for patients with SCM, which was consistent with the findings of previous studies. According to the multivariate Cox regression analysis, being older was independently linked to lower OS rates in patients, which was consistent with previous research on both SCM and general melanoma (20).Aging is correlated with physiological alterations in the organism, encompassing a deterioration in immune function. This could impede the body’s capacity to detect and counteract cancer cells, resulting in a less favorable prognosis for elderly melanoma patients. Melanoma is closely linked to prolonged exposure to ultraviolet (UV) radiation from sunlight throughout an individual’s lifespan (21). Advanced age typically entails greater cumulative sun exposure compared to younger counterparts, thereby heightening the susceptibility to melanoma development. Race and sex are associated with biological variations that can influence the development, progression, and treatment response of melanoma. Race and sex are critical prognostic factors for general cancer, and also for SCM; our results indicated that people who are male and black generally have poor OS rates, which was consistent with previous studies (2, 22).Sex hormones, namely estrogen and testosterone, possess the potential to influence the development and progression of melanoma. Moreover, genetic predispositions to melanoma may interact with environmental factors in disparate manners contingent upon race or sex, thereby exerting influence on the prognosis of the disease (23).

One of the prognostic factors included in the new model was marital status, which had not previously been found in a large-scale OS analysis. Whether marital status is a risk factor for SCM had not been reported previously; the current study found that being unmarried is a risk factor for survival (HR = 1.291, p < 0.01). According to a previous study, single people have a much higher risk of developing metastatic cancer, receiving inadequate care, and dying from the disease. Marital status and cancer prognosis may be linked for the following reasons: Firstly, married patients may have higher educational and financial statuses than single patients (24). Married people are also more likely to maintain therapeutic intervention regimens, which may affect how well they will recover. Secondly, a previous study found that married patients were less likely than single patients to present with metastatic disease (25). Finally, marriage was found to increase the likelihood of an early diagnosis of any form of cancer (26). In agreement with all of these previous observations, if a person is diagnosed with cancer when single, their probability of having an advanced form of the disease is higher, and their life expectancy is often shorter. In short, this study found that the prognoses for single patients are often not accurate, and single patients should be reminded of this more frequently.

Survival likelihood was also found to be affected by AJCC stage, regional node examination status, and treatment status. The AJCC stage is now commonly used to determine prognoses (27). The findings of the current study were consistent with the findings for general melanoma. Severe AJCC stages were positively associated with the survival rate.

Receiving radiation decreased the likelihood of survival in patients with SCM, which was consistent with the findings of previous studies. It should be noted that surgery is still the recommended treatment for SCM. Patients who have progressed to the point where surgery is no longer an option often receive radiation or chemotherapy (2, 28). Radiation therapy can induce adverse effects such as cutaneous irritation, lethargy, and impairment of adjacent viable tissues. In certain instances, these adverse reactions may surpass the anticipated therapeutic advantages, especially in scenarios where melanoma is not adequately managed, and resistance to radiation therapy may ensue (29). Consequently, tumor relapse or advancement may transpire despite the initial treatment response, thereby diminishing the prospects of survival. More experimental research into SCM is needed; however, because this was a retrospective cohort study, there were selection biases whose effects are difficult to identify. Further prospective studies are therefore needed to understand the exact relationship between radiation and SCM prognosis.

Patients who underwent regional node examination had better OS rates than those who did not. For patients with melanoma who are at high risk of regional node metastases, lymphatic mapping combined with sentinel lymph node biopsy is the recommended course of treatment (30). This approach can be used to identify patients who have positive sentinel lymph nodes and may benefit from adjuvant therapy. To correctly employ this strategy and ensure a low false-negative rate, significant experience is required to distinguish the sentinel lymph nodes (31).

**Figure 2:**
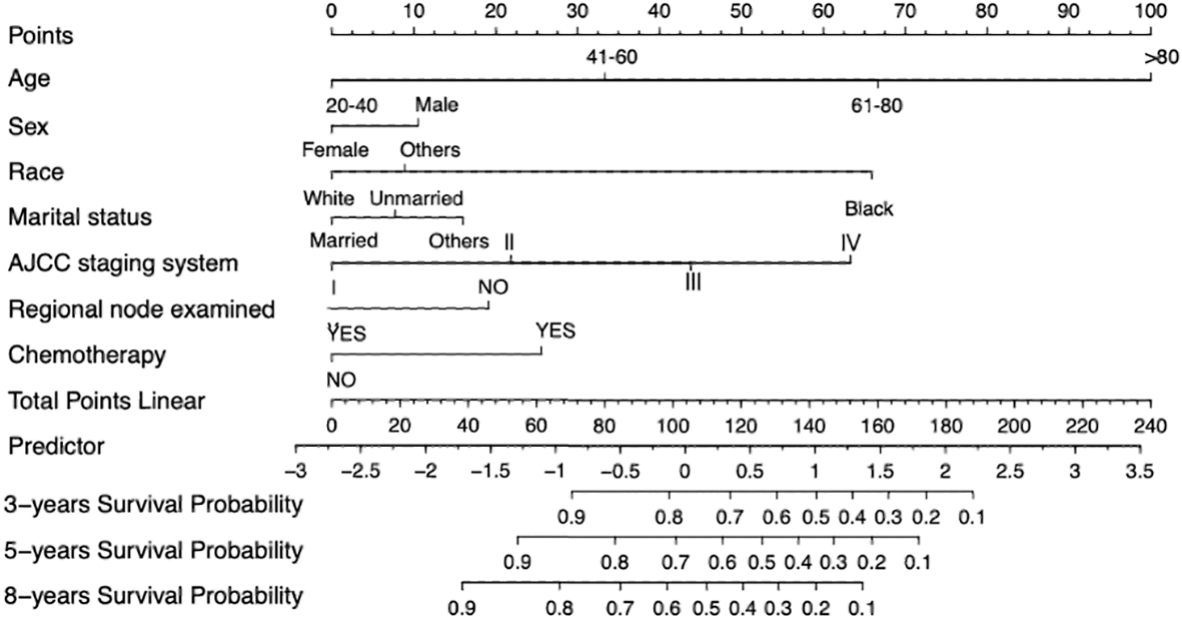
Nomogram predicting 3-, 5-, and 8-years OS probability.

**Figure 2** depicts the essential variables and how they affect the 3-, 5-, and 8-year OS probabilities of patients with SCM. This score can be used by healthcare practitioners to anticipate the likelihood of OS in individual patients, and then take steps that are more likely to improve their prognosis.

**Figure 3:**
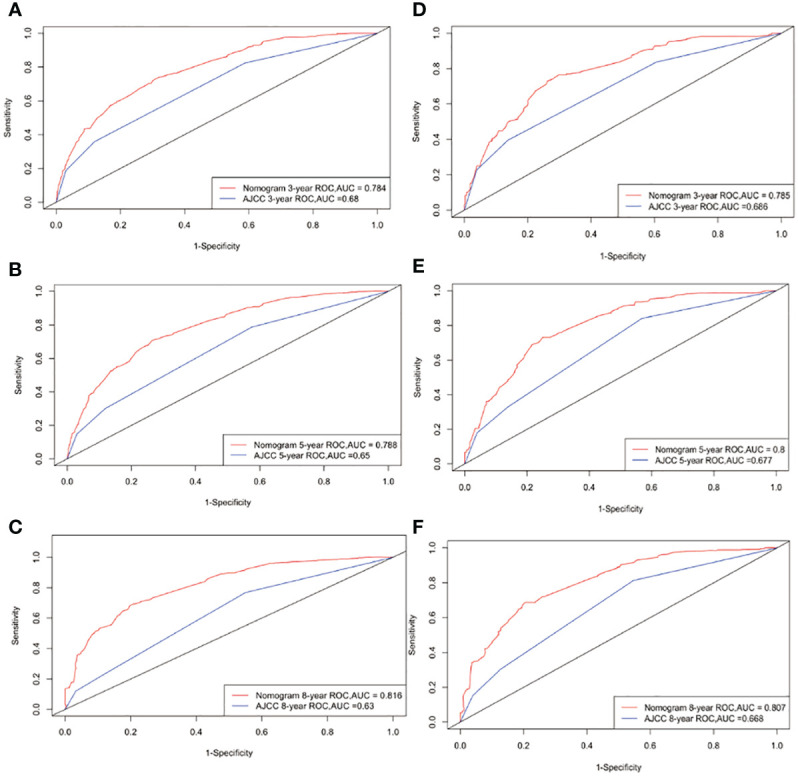
ROC curves. The area under the ROC curve (AUC) was used to evaluate the performance of the new nomogram. **(A–C)** represent the result of the training cohort; **(D–F)** represent the result of the validation cohort which compared to AJCC models.

Following the construction of the nomogram and the analysis of related prognostic markers, it was compared with the classic AJCC model using a training cohort and an internal validation cohort to evaluate the model underpinning the nomogram. We evaluated the discrimination performance using the C-index and AUC, and found that both were better for the nomogram than for the AJCC staging system in both the training and validation cohorts. **Figure 3** illustrates the ROC curves. The AUC was used to assess the performance of the new nomogram. Similarly, NRI is frequently used to evaluate the forecasting capabilities of two models, whereas IDI can be used to demonstrate overall model improvement (32, 33). The NRI of the prediction model indicated that after incorporating the new index, the proportion of correct classifications for the 3-, 5-, and 8-year survival probabilities increased by 62.5%, 68.0%, and 81.5%, respectively, in the training cohort, and by 61.6%, 74.2%, and 77.1% in the validation cohort (p < 0.001). The IDI indicated that the new model outperformed the previous model in terms of 3-, 5-, and 8-year survival probabilities by 15.1%, 18.3%, and 20.7%, respectively, in the training cohort, and by 13.1%, 16.6%, and 19.0% in the validation cohort (p < 0.001).

**Figure 4:**
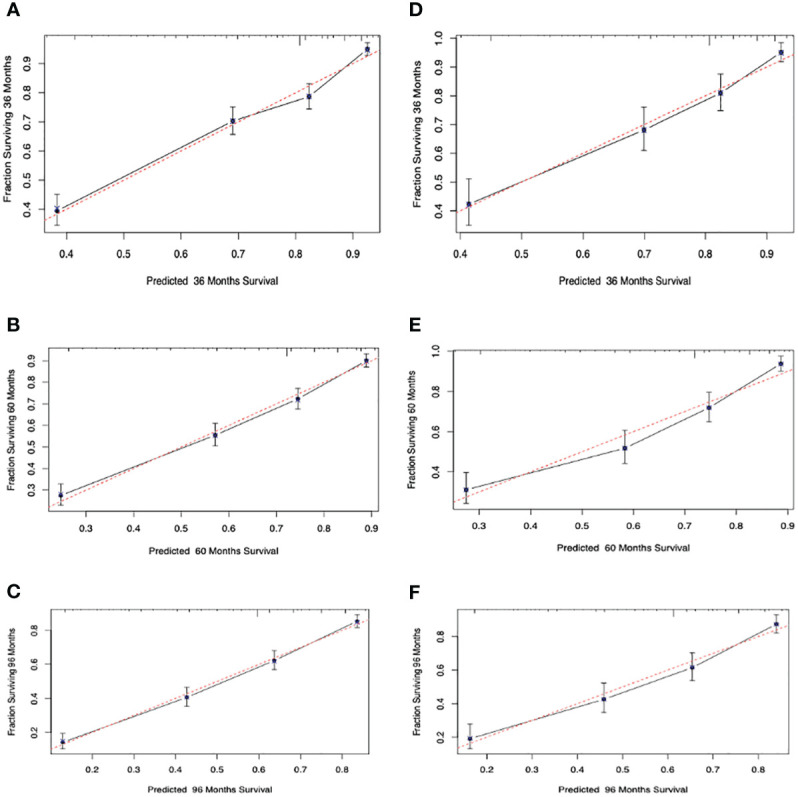
Calibration curves. Calibration curves for 3-, 5-, and 8-years cancer overall survival probability depict the calibration of each model in terms of the agreement between the predicted probabilities and observed outcomes of the training cohort **(A–C)** and validation cohort **(D–F)**.

Calibration curves were used to evaluate the calibration performance of the model. The 45-degree line indicates perfect calibration. The predicted dots in **Figure 4** are uniformly distributed, and the broken lines are exceptionally close to the standard line, indicating that the nomogram has strong calibration and discrimination abilities in both the training and validation cohorts.

**Figure 5:**
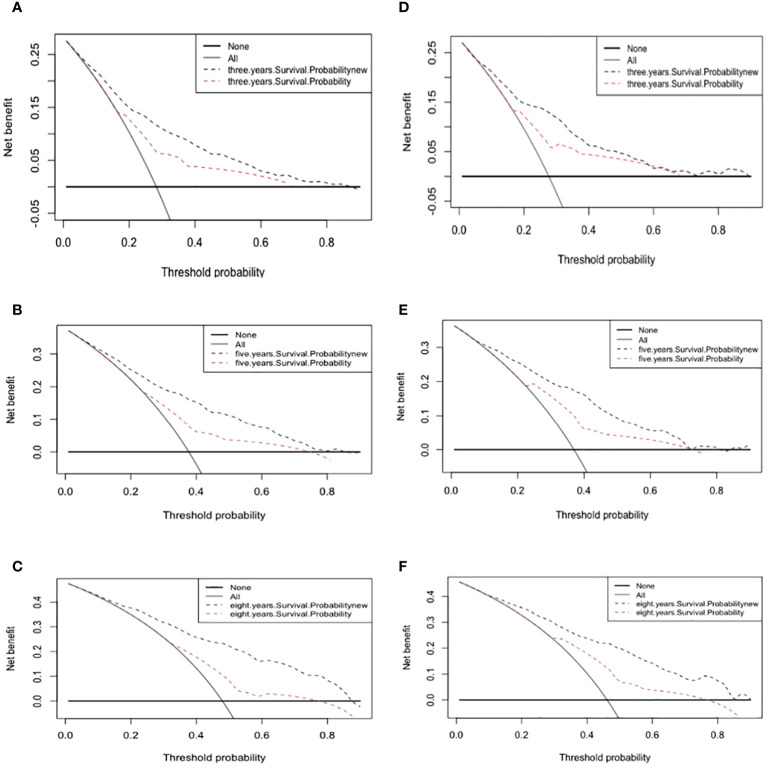
Decision curve analysis curves. Decision curve analysis of the training cohort **(A–C)** and validation cohort **(D–F)** for 3-, 5-, and 8-years cancer specific survival probability.

DCA is a method used to evaluate prediction models by determining their clinical net benefit (34, 35). **Figure 5** indicates that the DCA curves of the nomogram for 3-, 5-, and 8-year survival probabilities were almost all higher than those for the standard AJCC model, demonstrating the greater clinical efficacy of the new model.

### 4.1 Limitations

There were certain advantages to the inclusion of a large population in this study, but some drawbacks must also be considered. The retrospective design and use of the SEER database for data collection in the study undoubtedly led to selection and information biases (36); for example, combining “no” and “unknown” into one category in the SEER database may have produced incorrect results. The second limitation was that this study was not sufficiently detailed in some potentially major aspects; for example, specific biological indicators and behavioral patterns were not included. Finally, the nomogram was not validated externally, and using only internal validation may have caused overfitting of the new model. To obtain more-accurate results, we intend to add more predictors and evaluate the impact of the model using external cohorts in the future.

## 5 Conclusions

The SEER database was used in this study to construct a comprehensive nomogram for SCM, which was then evaluated using several metrics. Using a large population-based data set, we constructed and validated a nomogram for calculating the 3-, 5-, and 8-year OS rates in patients with SCM. These nomograms were found to be reliable, and our new nomogram can be utilized as a tool to assist clinical staff in predicting the 3-, 5-, and 8-year OS probabilities of patients with SCM more accurately than using the AJCC staging system. The nomograms can provide reference data for physicians to utilize when choosing individualized treatment options and delivering personalized prognoses because they are accurate and simple to use.

## Data availability statement

The datasets presented in this study can be found in online repositories. The names of the repository/repositories and accession number(s) can be found in the article/supplementary material.

## Author contributions

WL: Writing – original draft, Validation, Visualization, Data curation, Formal analysis, Software. LH: Writing – review & editing. XZ: Writing – review & editing. W-KM: Writing – review & editing. NL: Writing – review & editing. YT: Writing – review & editing. ZY:Writing – review & editing. HY: Writing – review & editing, Methodology, Supervision. JL:Writing – review & editing, Supervision, Project administration. LD: Writing – review & editing, Funding acquisition, Project administration.
